# Evaluation of iNSiGHT VET DXA (Dual-Energy X-ray Absorptiometry) for assessing body composition in obese rats fed with high fat diet: a follow-up study of diet induced obesity model for 8 weeks

**DOI:** 10.1186/s42826-019-0004-2

**Published:** 2019-06-24

**Authors:** Jungyun Yeu, Han-Jik Ko, Doyeun Kim, Youngbok Ahn, Jaejin Kim, Wonhee Lee, IlSin Jung, Jungkyu Suh, Seok-Jong Lee

**Affiliations:** 1 Osteosys, Seoul, Korea; 2 Woojung Bio, Suwon, Korea

**Keywords:** DXA, Body composition, Fat weight, Lean weight, Rat, Follow-up study

## Abstract

We examined the precision, accuracy, and capability of detecting changes of Dual-Energy X-ray Absorptiometry (DXA) for the measurements of total-body weight (TBW), total-body fat weight (TBFW), and total-body lean weight (TBLW) in an 8-week follow-up study of rats. Twenty male rats (4-week) were divided into 2 diet groups. For 8 weeks, we measured body composition (TBW, TBFW, TBLW) by DXA and TBW by an electronic scale once a week. In week 8, we measured body composition 5 times by DXA and TBFW by dissecting experiment (EXP) of euthanized rats (12-week). Total-body fat ratio (TBFR) was defined as TBFW/(TBFW+TBLW). The precision of DXA was evaluated by measuring the coefficient of variation (CV) and accuracy was evaluated by comparing DXA-derived data with EXP data. The capability of detecting changes of DXA in follow-up study was verified by analyzing the trend of DXA-derived values over the 8 weeks. For TBW, TBFW, TBLW of DXA, CVs were 0.02 ± 0.01, 0.10 ± 0.05, 0.03 ± 0.02 and errors were − 6.996 ± 3.429 (*r* = 0.999), + 14.729 ± 3.663 (*r* = 0.982), − 21.725 ± 4.223 (*r* = 0.991), respectively. Prediction models were [EXP TBW = − 31.767 + 1.085 (DXA TBW), R2 = 0.998, root mean square error (RMSE) = 1.842] and [EXP TBFR = − 0.056 + 1.177 (DXA TBFR), R2 = 0.948, RMSE = 0.007]. Over 8 weeks, DXA TBW and DXA TBLW steadily increased, DXA TBFW steadily increased followed by saturation or declination, difference of DXA TBFW between 2 diet groups steadily increased. In conclusion, our study verified that DXA (iNSiGHT VET DXA, OsteoSys, Korea) is accurate and precise enough to measure body composition of rats. Additionally, we confirmed the possibility that DXA could be used for the long-term follow-up studies.

## Introduction

The World Health Organization (WHO) has acknowledged obesity as one of the most obvious public health problems, and the prevalence of obesity has almost doubled globally in 2008 compared to 1980. Obesity causes many health-related problems either independently or in association with other diseases; particularly with type 2 diabetes, cardiovascular disease, the upward trend of certain cancers, respiratory complications, and arthritis. Therefore, obesity shortens life span associating with an increased diseases risk, and it was reported that obesity shortens almost 7 years of life span for 40 years old population [1].

Studies preparing or treating obesity are actively in progress including clinical trials mainly conducted on mice and rats to analyze the effect of food, medications, and exercise on obesity. In most of these studies, the degree of increase in actual fat weight was estimated with the fat tissue extracted by dissecting experiment [2–5]. Dissection experiment (EXP) method of a euthanized animal can be relatively accurate compared with the noninvasive methods. However, EXP method is a limited approach as it cannot estimate the longitudinal change of the fat weight while continuously observing the growth of live animal.

Dual-energy X-ray Absorptiometry (DXA) instrument can be applied as an option for longitudinal observations of body composition without killing animals. DXA is considered to be a gold standard for measuring bone density and body composition in the human body [6], and studies are being conducted to utilize it in the field of animal experiments as well [7–9].

In this study, we conducted an 8-week follow-up study using iNSiGHT VET DXA (OsteoSys, Korea) on the diet-induced obese rats fed with a high-fat diet and the rats fed with a normal diet observing the change of total body weight, fat weight, and lean weight. The accuracy of total body weight was verified with an electronic scale that was used weekly alongside with DXA. After the feeding period, the main fat tissues of the euthanized rats were extracted to verify the accuracy of DXA-derived fat weight by comparing the weights of the extracted fat tissues with the DXA-derived values. In addition, the data of five-time DXA scans without repositioning were analyzed for each rat to verify the basic precision of the DXA instrument.

## Methods/experimental

### Animals

A total of 20 male *Sprague Dawley* rats were the subjects of the study. At the beginning of the study, all rats were 4 week’s old and divided into two groups of 10 rats. Each group was fed with a high-fat diet (HFD, 45% kcal high-fat diet) and with a normal diet (ND, + 40 RMM-SP-10 Irradiated complete diet) respectively for the 8-week period.

The temperature of the animal room was maintained at 22 ± 2 °C, the relative humidity was 50.0 ± 15.0%, the illumination time was 12 h / day, the illumination was 150 ~ 300 LUX, and the ventilation frequency was 10 ~ 20 times / hour.

All process was carried out in accordance with the internal regulations of the animal ethics committee in Woojung Bio. IACUC approval number of animal experiment for this paper is WJIACUC20180731–3-11.

### Procedure

During the feeding period, Total body Weight (TBW), Total Body Bone Weight (TBBW), Total Body Fat Ratio (TBFR), Total Body Fat Weight (TBFW) and Total Body Lean Weight (TBLW) were measured weekly by cone beam flat panel detector DXA (iNSiGHT VET DXA, Osteosys, Korea), and TBW was measured with an electronic scale (Entris, sartorius, Germany).

After 8 weeks of feeding, CO2 gas was used for euthanasia, and then all rats was scanned by DXA instrument five times without repositioning (1-min rest between measurements) and their TBW was measured with an electronic scale. The electronic scale was also used to measure the weight of the main fat tissues that were gained by dissecting experiment (EXP). The main fat tissues were extracted from subcutaneous fat, epididymal adipose tissue, kidney adipose tissue, and intestine adipose tissue. The EXP TBFW was calculated by [EXP TBFW = sum of the weight of the extracted major fat tissues]. Since the TBBW was not measured separately in the dissection, it was also assumed that the DXA-derived TBBW was a true value. Therefore, the EXP TBLW was determined by [EXP TBLW = (EXP TBW - EXP TBFW) - DXA TBBW].

**Fig. 1:**
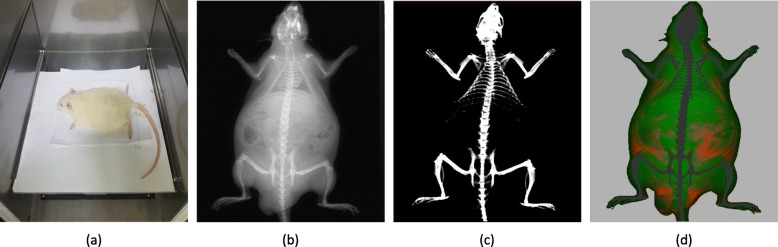
A sample rat and its image. **a** A rat placed on the scanning table of the instrument (dual x-ray absorptiometry; DXA), **b** x-ray image, **c** bone enhanced image, **d** color composition image

Until week 7, DXA measurements were performed on the rats that were sedated with gas inhalation (isoflurane) without supplemental anesthesia. Sedated rats were then placed on the scanning table with the prostrate position. DXA measurements, including 10 s of radiation output, took about 25 s per measurement. With the software provided by the manufacturer, bone areas were excluded from the collected X-ray images (Fig. 1), and then the body composition values were calculated by the embedded DXA algorithm.

To verify the accuracy, we calculated the differences and correlations between DXA-derived values and those obtained by dissecting experiment (EXP) on the euthanized rats. And then, the mean values and standard deviation of the errors of TBW, TBFW and TBLW were collected in all rats and the correlation values between the two measurement data were confirmed. In addition, a linear equation model predicting EXP values based on DXA-derived values were determined, and the R2 value was calculated.

The possibility of follow-up studies using DXA equipment was examined by comparing TBW and DXA TBW in regard to the mean value of all rats in the period of the 8 weeks. It was also examined whether the characteristics of each group changed consistently according to the intended experimental design by observing the changes of the mean TBFW of obesity-induced group and TBFW of the control group.

To verify the performance of DXA equipment, two quality control phantoms supplied with the instrument were measured weekly to collect fat ratio before a clinical test.

## Results

The precision of the DXA instrument was verified by the coefficients of variation (CV) of the repeated DXA analysis in Week 8. The mean coefficient of variation was lowest for DXA TBW of 0.02% and the highest for DXA TBFW of 0.10% (Table 1). The weekly change for fat ratio of two quality control phantoms (QP-ANI-1, QP-ANI-2) were 0.54 and 0.99% respectively for CV (Table 2).

**Table 1:** Basic precision of DXA values. The short-term precision of Dual-Energy X-ray Absorptiometry (DXA) calculated values in week 8 (*n* = 20 rats)

Contents	CV(%)^a^
DXA TBW (g)	0.02 ± 0.01 (0.01–0.04)
DXA TBFW (g)	0.10 ± 0.05 (0.03–0.18)
DXA TBLW (g)	0.03 ± 0.02 (0.01–0.06)

^a^The coefficient of variation are shown as mean ± SD with the range shown in parentheses

**Table 2:** Precision of DXA values for fat content of phantom. The precision of fat percentage (fat %) of quality control phantom measured by Dual-Energy X-ray Absorptiometry (DXA) for 8 weeks

Contents	CV(%)^a^
Fat % of QP-ANI-1 (%)	0.54 (30.51 ± 0.17)
Fat % of QP-ANI-2 (%)	0.99 (32.05 ± 0.32)

^a^The coefficient of variation are shown as mean with the mean ± SD of fat % shown in parentheses

**Fig. 2:**
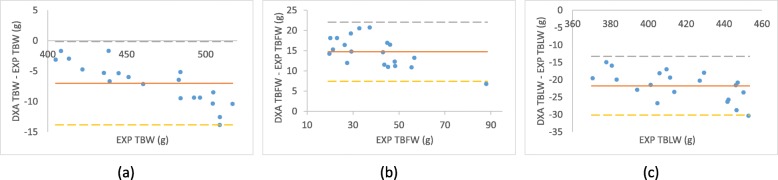
Residual plots of DXA values in week 8. Residual plots of DXA-derived values in week 8 for **a** total body weight (TBW), **b** total body fat weight (TBFW), and **c** total body lean weight (TBLW). The filled circles show the errors with dissecting experiment (EXP) for each rats with the solid line being the mean difference. The large dashed lines represent the mean ± 2 SD

The accuracy of the DXA instrument was first verified by comparing the data from dissecting experiment and those from DXA measurement in week 8(Table 3) and (Fig. 2). Compared to the EXP values, DXA TBW was underestimated (− 6.996 ± 3.429, *r* = 0.999), DXA TBFW was overestimated (14.729 ± 3.663, *r* = 0.982) and DXA TBLW was underestimated (− 21.725 ± 4.223, *r* = 0.991). In short, the DXA instrument estimated 98% of EXP TBW, 137% of EXP TBFW, and 95% of EXP TBLW.

**Table 3:** Accuracy of DXA values in week 8. The accuracy and Pearson correlations of DXA-derived composition values of rats in week 8

Contents	DXA^a^	EXP^b^	(DXA – EXP)^c^	Correlation coefficient ^d^
TBW (g)	458.205 ± 34.542 (401.879–506.719)	465.2 ± 37.507 (405–517.1)	−6.996 ± 3.429 (− 13.842 - -1.676)	0.999, *p* < 0.001
TBFW (g)	54.019 ± 14.324 (33.975–94.748)	39.29 ± 16.445 (19.722–87.955)	14.729 ± 3.663 (6.794–20.749)	0.982, *p* < 0.002
TBLW (g)	395.128 ± 24.603 (351.035–426.637)	416.852 ± 26.984 (370.567–452.873)	−21.725 ± 4.223 (− 30.317 - -14.927)	0.991, p < 0.001
TBBW (g)	9.059 ± 0.858 (7.723–10.169)			

^a^The DXA-derived values are shown as mean ± SD with the range shown in parentheses

^b^The EXP values are shown as mean ± SD with the range shown in parentheses

^c^The errors of DXA-derived value from EXP value are shown as mean ± SD with the range shown in parentheses

^d^The Pearson correlation coefficient value for DXA-derived values and EXP values calculated using “t-Test: Paired Two Sample for Means” in Excel

**Fig. 3:**
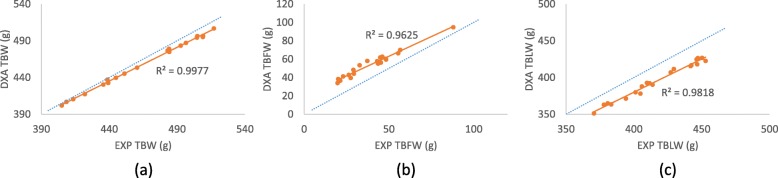
Relationship between DXA and EXP values in week 8. Scatter plots of the relationship of the values between DXA and EXP in week 8 for **a** TBW, **b** TBFW, and **c** TBLW. The small dashed lines represent the point where the values are same. The solid lines represent the trends of their relationship with R-squared values

It was confirmed that all EXP values and DXA-derived values (DXA TBW, DXA TBFW, and DXA TBLW) were highly correlated of R2 > 0.95 (Fig. 3), and based on this, we derived a regression equations for predicting EXP values from DXA-derived values (Table 4). EXP TBW can be predicted by DXA TBW [EXP TBW = − 31.767 + 1.085(DXA TBW), model R2 = 0.998, root mean square error (RMSE) = 1.842 g]. EXP TBLW and EXP TBFW were predicted by [EXP TBLW = (EXP TBW - EXP TBBW) × (1 - EXP TBFR)], and [EXP TBFW = (EXP TBW - EXP TBBW)] respectively. As EXP TBBW were not measured by dissection, we assumed EXP TBBW as [EXP TBBW = (DXA TBBW)]. Therefore, EXP TBFR can be estimated from DXA TBFR by [EXP TBFR = − 0.056 + 1.177 (DXA TBFR), model R2 = 0.948, root mean square error (RMSE) = 0.007].

**Table 4:** Prediction equations for EXP values. The prediction equation for the dissecting experiment (EXP) values from Dual-Energy X-ray Absorptiometry (DXA) derived composition values in week 8

Contents	Regression equations^a^	Model R2^b^
EXP TBW (g)	−31.767 + 1.085 (DXA TBW)	0.998
EXP TBFR (%)	−0.056 + 1.177 (DXA TBFR)	0.948
EXP TBBW (g)	DXA TBBW	^c^

^a^“Least squares” method was used

^b^The coefficient of determination for estimating regression equation

^c^This model for ‘EXP TBBW’ is the assumption

**Fig. 4:**
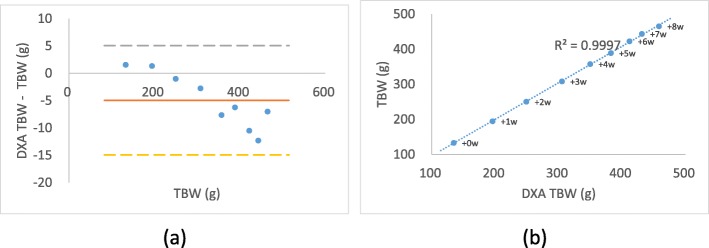
Residual plot and relationship between DXA and EXP values for 8 weeks. **a** Residual plot of DXA-derived TBW values for 8 weeks. Each filled circle show the mean error of all rats (*n* = 20) with the values measured by an electronic scale for each weeks with the solid line being the mean difference. The large dashed lines represent the mean ± 2 SD. **b** Scatter plot of the relationship of mean TBW (*n* = 20) between DXA and an electronic scale for 8 weeks with the labels represented each weeks. The small dashed line represents the point where the values are same. R-squared value of their trend line is also represented in graph

**Fig. 5:**
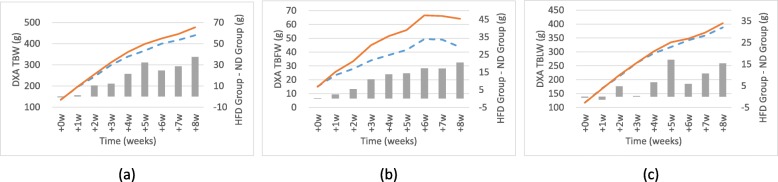
Trend graph of DXA values for 8 weeks. The graphs represent the trend of mean DXA values divided into the high fat diet (HFD) group and the normal diet (ND) group for 8 weeks for (**a**) TBW, (**b**) TBFW, and (**c**) TBLW. The solid lines are the trend of HFD group and small dashed lines are the trend of ND Group. Bar graph means the difference between two groups

The accuracy of the average DXA TBW for a total of 8 weeks was weekly verified by the average TBW measured with an electronic scale (Table 5) and (Fig. 4). Compared to TBW, DXA TBW was underestimated (− 4.951 ± 5.008, r = 0.999). During the study period, the rats were divided into high-fat diet (HFD) group and normal diet (ND) group, and the changes of DXA-derived values (DXA TBW, DXA TBFW, and DXA TBLW) of these two groups were confirmed (Fig. 5). DXA TBW and DXA TBLW increased steadily in both groups from the beginning (week 0) to the end of the study (week 8). DXA TBFW increased steadily followed by saturation or declination at week 6 in both groups. However, the difference of DXA TBFW between the two groups showed increasing tendency until week 8.

**Table 5:** Accuracy of DXA values for 8 weeks. The accuracy and Pearson correlation of Dual-Energy X-ray Absorptiometry (DXA) derived composition values with the electronic scale values of rats for 8 weeks

	DXA^a^	Electronic scale^b^	(DXA - Electronic scale)^c^	Correlation coefficient^d^
TBW (g)	324.248 ± 111.581 (134.508–458.205)	329.198 ± 116.164 (132.945–465.2)	−4.951 ± 5.008 (− 12.312–1.563)	0.999, *p* < 0.009

^a^The DXA-derived values are shown as mean ± SD with the range shown in parentheses

^b^The electronic scale values are shown as mean ± SD with the range shown in parentheses

^c^The errors of DXA-derived value from the electronic scale value are shown as mean ± SD with the range shown in parentheses

^d^The Pearson correlation coefficient value for DXA-derived values and the electronic scale values calculated using “t-Test: Paired Two Sample for Means” in Excel

## Discussion

### Precision

The precision of DXA instrument can be affected by several factors. First, it is possible that a variation of the values results from the movement when the subject is breathing under anesthesia, or from the changed position and posture when the subject is placed on the scanning table of the instrument. The electrical noise of hardware such as X-ray tubes and detectors included in DXA instrument and the variation caused by errors in image processing or calculation algorithms for values in software also can affect the precision. In addition, environmental factors such as temperature and humidity, and changed hardware property can be considered as a variation factor affecting the study under a long period of time.

The precision of the DXA instrument to be verified in this study was for the short-term repeated measurement at the same position for each subject. We assumed that this was the most basic variation factors be confirmed before considering the effect of the movement or of the changed position/posture by the subject. Especially, if the precision of the short-term repeated measurement is high, precise results can be obtained without additional measuring of the same subject nor increasing the measuring time.

The coefficient of variation (CV) calculated from the sample standard deviation (*n* = 5) of DXA TBW, DXA TBFW, and DXA TBLW after euthanasia was highest in average of DXA TBFW (*n* = 20) of 0.10%, and the maximum value was 0.18% (Table 1). Considering the average DXA TBFW which was 54.019 g (Table 3), the standard deviation between the short-term repeated measurements of the DXA instrument used in this study could be verified with significantly high precision to the degree of 0.054 g from the DXA TBFW value.

In addition, the precision of long-term repeated measurements for the DXA instrument was also verified. For this purpose, the quality control phantoms provided with the DXA instrument was measured once a week before the test to check the range of fat percentage (fat %) and CV (Table 2). The reason for applying fat percentage corresponding to TBFR, instead of directly verifying TBW, TBFW, or TBLW, was because there was the difference between the weight of phantom and those of the rats. As the fat percentage ranges from 0 to 100% regardless of the weight of the individual, it can be used to estimate the variation in DXA TBFW in the rats resulted from the changed hardware property over long-term repeated measurements.

For the two quality control phantoms, the CV of fat percentage calculated from the weekly measured data for 8 weeks was 0.54 and 0.99%. That is, the precision of the fat percentage was controlled within 1% CV during the test. In the QP-ANI-2 phantom, the mean value of fat percentage was 32.05% and the standard deviation was 0.32%. Considering the average weight of the rats (DXA TBW - DXA TBBW) which was 449.146 g (Table 3), it could be estimated that the standard deviation of DXA TBFW or DXA TBLW resulted from the changed hardware property over the test period was 1.437 g in maximum.

### Accuracy

To verify the accuracy, it is mandatory to have a reference value for comparison. In this study, the main fat tissue was extracted through a dissecting experiment (EXP) to verify the accuracy of the body composition of the rats measured by the DXA instrument. We assumed the sum of the weight of fat tissues measured by the electronic scale as the total fat weight (TBFW). We also measured the total weight (EXP TBW) of the rats prior to dissection and assumed that DXA TBBW was true because the bone mineral contents corresponding to EXP TBBW were not measured. Using these values, the lean weight (EXP TBLW) of the rats was calculated and used as a reference value. All rats (*n* = 20) were euthanized after 8 weeks of follow-up and then dissections were performed. The values measured from dissections were compared with the DXA-derived values to verify the accuracy of the DXA instrument.

DXA TBW was underestimated with the error of − 6.996 ± 3.429 g compared to EXP TBW (Table 3). It was lower about 1.53% than the average weight of 458.205 g, which seemed to be affected by the fact that the tail was not entirely included in the measurement image of the rat, rather than the error of the DXA instrument. The imaging area of this DXA instrument was sufficient to include the entire body of the rat, but part of the tail was not included in the image when the posture of the rat was fully prostrated (Fig. 1). Given this point, we could conclude that the weight estimating performance of this DXA instrument for the measurable areas was even more accurate than the obtained results.

DXA TBFW and DXA TBLW were estimated by DXA software from DXA TBW, DXA TBBW and DXA TBFR. DXA TBFW was overestimated with the error of 14.729 ± 3.663 g compared to EXP TBFW and DXA TBLW underestimated with the error of − 21.725 ± 4.223 g. In other words, there was a difference between the DXA TBFR and the EXP TBFR. The algorithm in the DXA software might have caused the difference, but the fat tissues located other than the ones extracted from the dissection could be regarded as the possible reason. For more accurate measurement and verification for fat mass, there was the chemical analysis called Carcass method [9], but we excluded this method in this study from the perspective of time, process, and budget requested for the analysis of the method.

Due to the strong correlation (R2 > 0.95) (Fig. 3), it was suggested that this DXA instrument was accurate enough to predict the EXP values while there were errors between EXP values and DXA-derived values in TBFW and TBLW. Therefore, we derived a simple linear equation to predict the EXP values using DXA-derived values. As the ratio of DXA TBFW to DXA TBLW determines the DXA TBFR, all values can be estimated by DXA TBFR. Therefore, we only estimated the relationship between EXP TBW and EXP TBFR and DXA-derived values. The R2 values of the derived equation for EXP TBW was 0.998 and for EXP TBFR was 0.948. Because R2 from both predictions were higher than 0.9, the equations by DXA measurements would be a simple substitute if the similar dissections are requested in the other experiment.

The another purpose of this study was to evaluate whether the difference in body composition of high-fat diet (HFD) group and that of normal diet (ND) group can be accurately estimated by DXA instrument.

Because of the impossibility to conduct the dissections during the 8-weeks feeding period, it was TBW by the electronic scale for each rat that could be collected only during the 8-week period to be compared directly with DXA-derived values. However, by comparing the TBW and the DXA TBW, it was confirmed that the weight of the rats were estimated very accurately as the result of dissection in week 8. The average of error of the DXA TBW (*n* = 20) was − 4.951 ± 5.008 g compared to the TBW (Table 5), and the accuracy of estimating the actual weighing scale with DXA TBW was R2 = 0.999 (Fig. 4).

Analysis of the results from the dissection in week 8, it was confirmed that DXA-derived values (TBW, TBFW, and TBLW) estimated with high accuracy, and also confirmed that weekly measured DXA TBW until week 8 was accurate. Therefore, we could estimate that DXA TBFW and DXA TBLW until week 7 would be reliable enough as well.

Another context in support of this estimation was confirmed by dividing DXA-derived values into the HFD Group and the ND Group (Fig. 5). The difference between the two groups in the DXA TBFW showed a steady increase over the test. Finally, in the last week 8, we found a similar difference between the two groups in the TBFW and the TBFW even though TBFW was measured with lower values compared to TBLW. The TBFW difference between the two groups was significantly larger (20.50 g at week 8) than the estimated variation (1.450 g) by the changed condition of the instrument during the test period. Therefore, it was possible to confirm that the high-fat diet has a clear effect on the fat gaining process. TBW and TBLW continued to increase while TBFW decreased in both groups from week 6. Nonetheless, the increasing tendency in the differences between the two groups supported the assumption that the DXA instrument would be able to estimate even small changes in the rat.

## Conclusions

Based on the abovementioned review of the accuracy, we can suggest that follow-up test for measuring the change of fat weight and lean weight can be conducted without extra dissection if the test is prepared with the DXA instrument.

The limitation of this paper is that no direct validation of other body composition values of DXA except the TBW value was performed during the lifetime of the rat. Therefore, in order to overcome this limitation, it will be necessary to compare with other validated instruments or to perform weekly dissecting experiment with a sufficient number of animals.

## Abbreviations

CV: Coefficient of Variation
DXA: Dual-Energy X-ray Absorptiometry
EXP: Dissecting Experiment
HFD: High Fat Diet
ND: Normal Diet
RMSE: Root Mean Square Error
TBBW: Total Body Bone Weight
TBFR: Total Body Fat Ratio
TBFW: Total Body Fat Weight
TBLW: Total Body Lean Weight
TBW: Total Body Weight
WHO: The World Health Organization
